# Matching sensor ontologies with unsupervised neural network with competitive learning

**DOI:** 10.7717/peerj-cs.763

**Published:** 2021-11-19

**Authors:** Xingsi Xue, Haolin Wang, Wenyu Liu

**Affiliations:** 1 Intelligent Information Processing Research Center, Fujian University of Technology, Fuzhou, Fujian, China; 2 Guangxi Key Laboratory of Automatic Detecting Technology and Instruments, Guilin University of Electronic Technology, Guilin, Guangxi, China; 3 School of Computer Science and Mathematics, Fujian University of Technology, Fuzhou, Fujian, China

**Keywords:** Artificial intelligence of things, Sensor ontology matching, Unsupervised neural network, Competitive learning

## Abstract

Sensor ontologies formally model the core concepts in the sensor domain and their relationships, which facilitates the trusted communication and collaboration of Artificial Intelligence of Things (AIoT). However, due to the subjectivity of the ontology building process, sensor ontologies might be defined by different terms, leading to the problem of heterogeneity. In order to integrate the knowledge of two heterogeneous sensor ontologies, it is necessary to determine the correspondence between two heterogeneous concepts, which is the so-called ontology matching. Recently, more and more neural networks have been considered as an effective approach to address the ontology heterogeneity problem, but they require a large number of manually labelled training samples to train the network, which poses an open challenge. In order to improve the quality of the sensor ontology alignment, an unsupervised neural network model is proposed in this work. It first models the ontology matching problem as a binary classification problem, and then uses a competitive learning strategy to efficiently cluster the ontologies to be matched, which does not require the labelled training samples. The experiment utilizes the benchmark track provided by the Ontology Alignment Evaluation Initiative (OAEI) and multiple real sensor ontology alignment tasks to test our proposal’s performance. The experimental results show that the proposed approach is able to determine higher quality alignment results compared to other matching strategies under different domain knowledge such as bibliographic and real sensor ontologies.

## Introduction

Artificial Intelligence of Things (AIoT), which integrates Internet of Things (IoT) systems with Artificial Intelligence (AI) technology, is rapidly developing in the field of wireless communication (Ghosh, Chakraborty & Law, 2018; Burhanuddin et al., 2018; Li, Xu & Zhao, 2015; Lin et al., 2020). As an important part of AIoT, interconnected things with built-in sensors can sense their surroundings, collect, store, transmit and process relevant data (Kocakulak & Butun, 2017; Xu & Helal, 2015; Sung & Yang, 2013). Due to the large number of heterogeneous sensor nodes deployed, achieving trusted communication and collaboration between systems in AIoT is still one of the challenges (Sisinni et al., 2018; Sung & Yang, 2014). To face this challenge, it is necessary to use the semantic annotation of sensor data to integrate and share conceptual models, which necessitates the use of sensor ontology techniques (Wang, Zhang & Li, 2015; Xue & Pan, 2018). Sensor ontologies are able to formally model knowledge in AIoT through defining the core concepts and relationships between them, such as sensor capabilities, performance, accuracy, etc. (Liu et al., 2019; Huang, Xue & Jiang, 2020). However, due to the subjectivity of ontology building process, sensor ontologies might be defined by different terminologies and contexts, leading to the heterogeneity problem (Xue et al., 2021a). To solve this problem, it is important to determine the correspondences between sensor ontologies’ concepts, which is known as sensor ontology matching (Xue & Zhang, 2021; Xue & Chen, 2020).

In recent years, neural networks have been considered as the effective approach to solving the ontology alignment problem (Ardjani, Bouchiha & Malki, 2015; Khoudja, Fareh & Bouarfa, 2018b; Chen et al., 2020). However, the existing neural network-based sensor ontology matching methods suffer from two drawbacks (Anam et al., 2015): (1) they consider ontology matching as a regression problem that need to predefine a threshold to filter the erroneous correspondences, but its value is difficult to determine; (2) a large of training samples are needed to train the network, which is difficult to satisfy in practical applications. To overcome these two drawbacks, in this work, an unsupervised neural network model for aligning sensor ontologies is proposed, which uses the competitive learning approach to efficiently cluster the ontologies to be matched. In general, the main contributions of this work are listed as follows: (1) we formally construct a binary classification model for the ontology matching problem, which avoids predefining a threshold; (2) an unsupervised neural network with competitive learning is proposed, which is able to effectively solve the sensor ontology matching problem without the requirement of labelled training samples.

The rest of this paper is as follows: after introducing the neural network-based ontology matching techniques (“Neural Network Based Ontology Matching Techniques”), definitions of sensor ontology matching and various similarity measure techniques are given (“Preliminaries”). Then a binary classification model for the ontology matching problem is defined, and the competitive learning mechanism and network framework are presented (“Methodology”). Experiments and results analysis are then performed (“Experiment”). Finally, the conclusion is drawn and the future work is presented (“Conclusions”).

## Neural network based ontology matching techniques

Sensor ontology matching aims at determining the correspondences between heterogeneous entities in two sensor ontologies (Jiang & Xue, 2021; Xue et al., 2021b). Recently, various heuristic-based algorithms have been proposed to address the ontology matching problem, such as Genetic Algorithms (GA) (Xue & Chen, 2018), Particle Swarm Algorithms (PSO) (Lv, Jiang & Li, 2020) and Argumentation Frameworks (AF) (Xue et al., 2021a), and neural networks are one of the most popular approaches (Khoudja, Fareh & Bouarfa, 2018a; Rubiolo et al., 2012). The first application of neural networks in information integration was in 2000, when SEMINT was the first system to use neural network to implement semantic integration of heterogeneous databases (Li & Clifton, 2000). Hariri, Abolhassani & Sayyadi (2006) proposed a supervised learning-based neural network to integrate similarity measures. The network model is trained using ontology instances and sensitivity analysis is performed to select the optimal integration weights. Djeddi & Khadir (2012) proposed an artificial neural network algorithm to determine how to combine multiple similarity measures into one aggregated measure to improve ontology alignment quality. This is the first time a neural network is used to solve the ontology meta-matching problem. Alboukaey & Joukhadar (2018) argue that the integration of multiple similarity measures greatly affects the final quality of the matching system. To improve this point, the authors used a regression algorithm to automatically find multiple possible combinations of similar measures. Jiang & Xue (2020) introduced character embedding techniques to represent the semantic and contextual information of concepts and proposed an ontology matching technique based on long and short-term memory networks (LSTM). More recently, convolutional neural networks have been introduced in ontology matching to perform alignment between strings using character embeddings (Bento, Zouaq & Gagnon, 2020). Zhu, Zhang & Xue (2021) proposed a semi-supervised learning based sensor ontology matching technique in order to efficiently determine the correspondence of sensor entities. This approach requires the concept of “centrality” to construct training instances.

Existing neural network-based ontology matching techniques model the matching problem as a regression problem and use the threshold to filter the alignments. In addition, supervised learning-based neural networks require a large number of matched entities or instances as training samples to train the network. It is difficult to find labelled training sets in practical applications. To address these issues, an unsupervised neural network model is proposed in this work, which introduces a competitive learning mechanism to replace the traditional error backpropagation algorithm, and mapping the matching results to the output layer of binary classification.

## Preliminaries

### Sensor ontology matching

**Figure 1:**
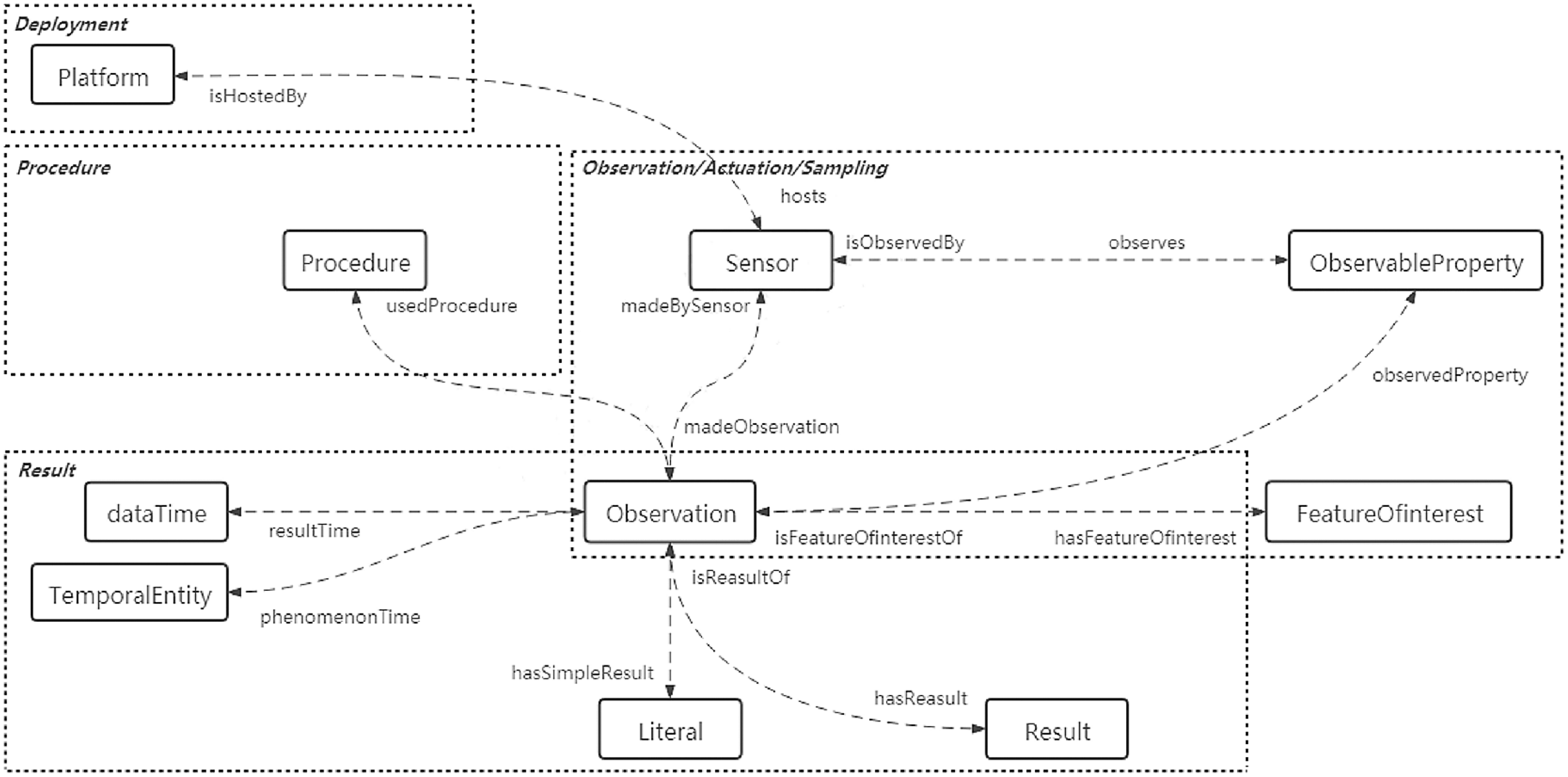
Overview of the SOSA partial classes and properties.

The sensor ontology normatively describes the core concepts and their relationships in the sensor domain (Xue & Chen, 2019), which can be defined as a 3-tuple (*C*, *OP*, *DP*), where *C* represents the set of classes, that is, the core concepts of the knowledge domain; *OP* is the set of object properties and *DP* is the set of data properties, both of which describe the relationships that exist between concepts (Xue & Wang, 2015). Figure 1 illustrates the classes and properties between the different concept modules of the SOSA (Sensor, Observation, Sample and Actuator) ontology, a lightweight core ontology designed for a pervasive target audience and sensor domain. More specifically, the rounded rectangles indicate classes and the dotted arrows indicate properties that describe the relationships between classes. The dotted boxes represent several conceptual modules covering key sensor, actuation and sampling concepts.

**Figure 2:**
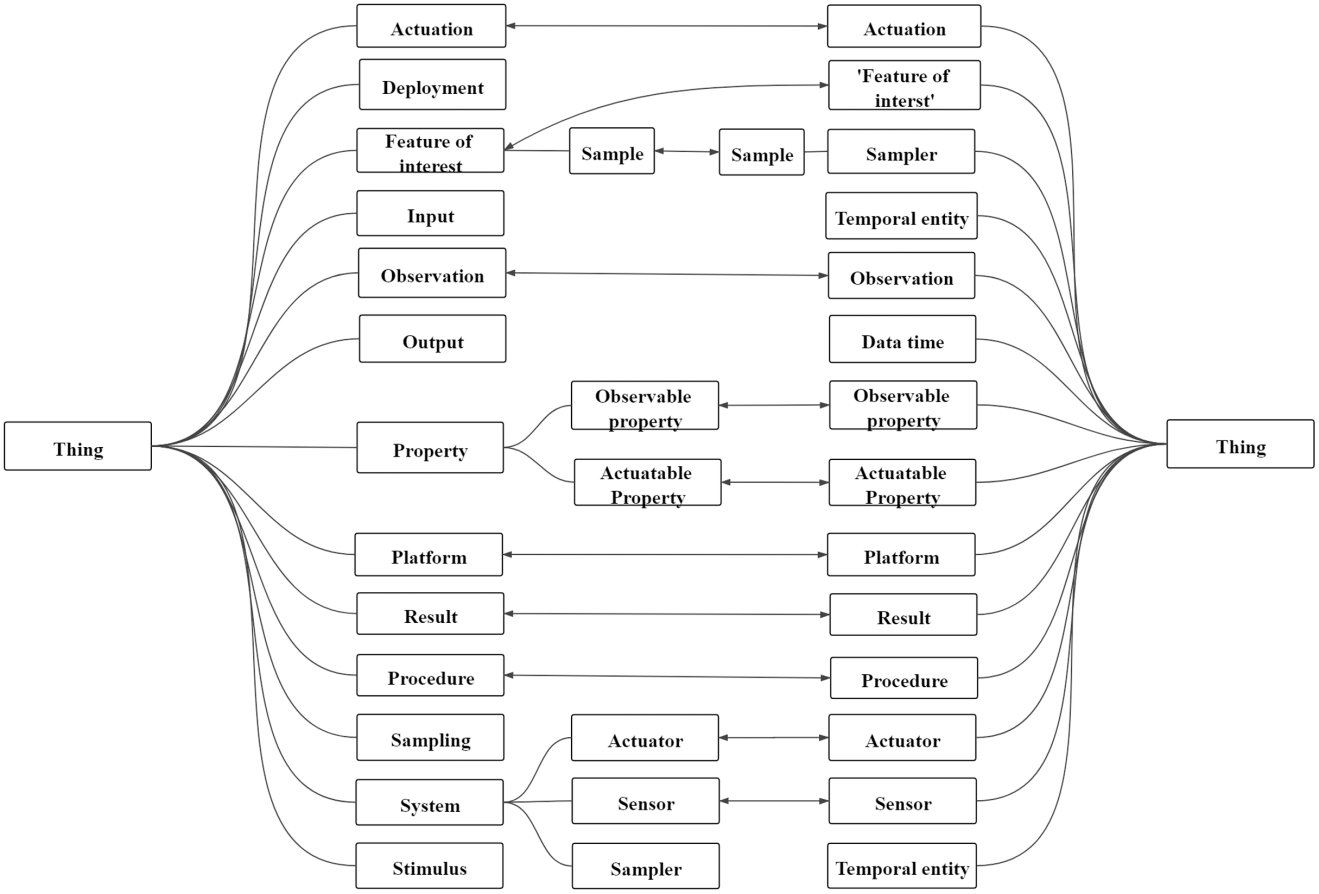
An example of sensor ontology matching.

Sensor ontology matching is about constructing relationships between sensors, bridging the semantic gap between them, and finding the same sensor entities (Xue & Liu, 2017). The ontology matching process is a function: *A* = *ϕ*(*O*_1_, *O*_2_, *P*, *RA*), where *A* is the final alignment; *O*_1_ and *O*_2_ are two ontologies to be matched; *P* is the experimental parameter; *RA* is the reference alignment (Chu et al., 2020; Xue, 2020). In general, the final alignment between sensor ontologies’ concepts are dichotomized into disjointness and equivalence. Figure 2 depicts an example of the alignment on two sensor ontologies. Furthermore, the rounded rectangle represents the entities in the sensor ontology. The connections between entities on the same side represent structured information, that is, superclass relationships between entities. The bidirectional arrows represent the mapping between the two entities, that is, equivalence correspondence, and all mappings between the two sensor ontologies are called sensor ontology alignment.

### Similarity measure

The similarity measure is a function that takes the entities of two ontologies as input and outputs a real number between (0, 1) to represent their similarity. Since no single similarity measure can guarantee robustness in any matching test, in general, to improve the accuracy of alignment results, sensor ontology matching systems aggregate multiple similarity measures. The subsequent sections describe the similarity measures used in this work.

Lexical-based similarity measure calculates the morphological similarity of two words. Four lexical-based similarity measures are adopted, that is, Jaro distance which uses the number and position of characters needed to convert two strings, Levenshtein distance which calculates the number of operations such as modification, deletion and insertion of characters, *n*-gram similarity which calculates the distance between two strings using the number of common substrings, and Dice coefficient which measures the similarity of strings. Given two strings *s*_1_ and *s*_2_, formulas are defined as follows:

(1) $$Jaro\,\,({s_1},{s_2}) = \displaystyle{1 \over 3} \times \left(\displaystyle{{com({s_1},{s_2})} \over {|{s_1}|}} + \displaystyle{{com({s_1},{s_2})} \over {|{s_2}|}} + \displaystyle{{com({s_1},{s_2}) - diff({s_1},{s_2})} \over {com({s_1},{s_2})}}\right),$$

(2) $$Levenstein\,\,({s_1},{s_2}) = \max \left\{ 0,\displaystyle{{min\{ |{s_1}|,|{s_2}|\} - trans({s_1},{s_2})} \over {min\{ |{s_1}|,|{s_2}|\} }}\right\} ,$$

(3) $${\it n{-}gram}\,\,({s_1},{s_2}) = \displaystyle{{|f({s_1},n) \cap f({s_2},n)|} \over {min\{ |{s_1}|,|{s_2}|\} - n + 1}},$$

(4) $$Dice\,\,({s_1},{s_2}) = \displaystyle{{2*com({s_1},{s_2})} \over {|{s_1}| + |{s_2}|}},$$

where |*s*_1_| and |*s*_2_| are the lengths of string *s*_1_ and *s*_2_, respectively; *com*(*s*_1_, *s*_2_) is the number of common characters of *s*_1_ and *s*_2_; *diff*(*s*_1_, *s*_2_) is the edit distance between *s*_1_ and *s*_2_; *trans*(*s*_1_, *s*_2_) is the number of common character pairs in different positions; *f*(*s*_*i*_, *n*) represents several substrings of *s*_*i*_ with length *n*. Based on empirical values, in this work, *n* is 2.

In addition to measuring the similarity of entities by lexical-based approaches, the semantic similarity between them can also be calculated by linguistics-based measures of similarity values, which determine two words’ similarity by considering their synonym or hypernym relationship. Given two words *w*_1_ and *w*_2_, their similarity is calculated using the WordNet electronic vocabulary database. The definition is as follows:

(5) $$WordNet\,\,({w_1},{w_2}) = \left\{ {\matrix{ {1,} \hfill  {{\rm if}\;{\rm two}\;{\rm words}\;{\rm are}\;{\rm synonymous,}} \hfill \cr {0.5,} \hfill  {{\rm if}\;{\rm two}\;{\rm words}\;{\rm are}\;{\rm the}\;{\rm hypernym,}} \hfill \cr {0,} \hfill  {{\hskip-7.3pc}{\rm otherwise}{\rm .}}}} \right.$$

Since this work utilizes neural networks as a classification model, as many similarity measure techniques as possible are chosen. Meanwhile, to ensure the quality of matching at different semantic levels, two types of measures are chosen, that is, lexical-based measures and linguistics-based measure.

## Methodology

### Ontology matching problem

The semantic correspondence between similar entities in two ontologies is called ontology alignment. According to the literature (Xue et al., 2021b), the ontology alignment can be defined as follows: Given two ontologies *O*_1_ and *O*_2_, the alignment between them is a set of correspondence: $\langle e1, e2, r\rangle$, where *e*_1_ ∈ *O*_1_, *e*_2_ ∈ *O*_2_ are matched entity pairs and *r* denotes the relationship between *e*_1_ and *e*_2_. The relationship is dichotomized into disjointness and equivalence, therefore the ontology matching problem can be transformed as a binary classification problem.

In this work, we construct a binary classification process for the ontology matching problem. First determine a similarity matrix *M* = [*m*_*ij*_] _|*O*1| * |*O*2|_, where |*O*_1_| and |*O*_2_| represent the cardinalities of two ontologies *O*_1_ and *O*_2_ respectively. For each pair of potentially aligned entities *e*_1_ and *e*_2_, their similarity is calculated by the multiple similarity measures mentioned above. In order to map the similarity to a binary decision, *m*_*ij*_ denotes the relationship between entities *e*_1_ and *e*_2_, corresponding to values 0 or 1, that is, *m*_*ij*_ = {0, 1}. Furthermore, the ontology matching problem is to investigate whether the two entities to be aligned are equivalent and evaluate the quality of the final alignment based on the reference alignment. For the best quality of the ontology alignment, the objective function is to maximize *f-measure* (*M*).

Neural networks are a multi-input connection model that has a widely application in classification problems (Chen, 2020). For the above mathematical model, in this work, we use unsupervised neural networks based on competitive learning to solve this binary classification problem.

### Competitive learning mechanism

Traditional neural network models require back propagation algorithms to adjust the network weights when dealing with classification problems, which inevitably requires a large number of labelled training samples to calculate the error. For the sensor ontology, the labelled training samples are matching pairs that have determined the relationship between them. To reduce the reliance on labeled samples and achieve the goal of unsupervised learning, we train the network using the competitive learning strategy, where the output neurons of the network compete with each other and only the winner neuron adjusts the weight vector, while the state of the other neurons is suppressed. The process does not require the expectation value to calculate the error and adjust the weights, therefore unsupervised learning can be achieved. In the next, the pseudo-code for competitive learning is shown by Algorithm 1.

**Algorithm 1:** The competitive learning procedure of the neural network.

**Require:** Training sample: *X*_*i*_ ∈ *R*^*p*^, *i* = 1,2,…,*n*; Number of network training: *epoch*; Learning rate: *η*
**Ensure:** Neural networks with connected weights
1: Initialize the network weight *W*;
2: **for** epoch **do**
3: Input vector normalization: *normalization*(*X*_*i*_);
4: Sigmoid as activation function: *o* = *sigmoid*(*dot*(*W*,*X*_*i*_));
5: Compute winner neurons: *winner* ← *where*(*max*(*o*));
6: Update winner neuron connected weights: *W*[*winner*] ←*W*[*winner*] + *η* * (*X − W*[*winner*]);
7: Weight normalization: *normalization*(*W*[*winner*]);
8: Update learning rate: *η ←η* − *η*/*epoch*;
9: **end for**

First, the training sample *X* is input to the network, and each similarity vector *X*_*i*_ is a p-dimensional real vector. The weight vectors corresponding to all neurons in the competitive layer are compared with *X*. The weight vector that is more similar to *X* is the winner neuron, and its weight is noted as *W*_j*_. Second, the winner neuron adjusts its weight, and the weights of other neurons remain unchanged. The weight vector adjustment strategy is as follows:

(6) $$\left\{ {\matrix{ {{{\bf W}_{{j^*}}}(t + 1) = {{\widehat {\bf W}}_{{j^*}}}(t) + \eta \left( {\widehat {\bf X} - {{\widehat {\bf W}}_{{j^*}}}} \right)\quad j = {j^*},} \hfill \cr {{{\bf W}_j}(t + 1) = {{\widehat {\bf W}}_j}(t)\quad j \ne {j^*},} \hfill \cr } } \right.$$

where *η* is the learning rate, which is used to adjust the weights and generally decreases with the iteration of the algorithm. j* represents the winner neuron and *t* represents the generation of the algorithm. After normalization of the weight vector, the algorithm enters the next generation. When the algorithm terminates, the network training ends and the network connected weights are determined.

### The framework of unsupervised neural networks

**Figure 3:**
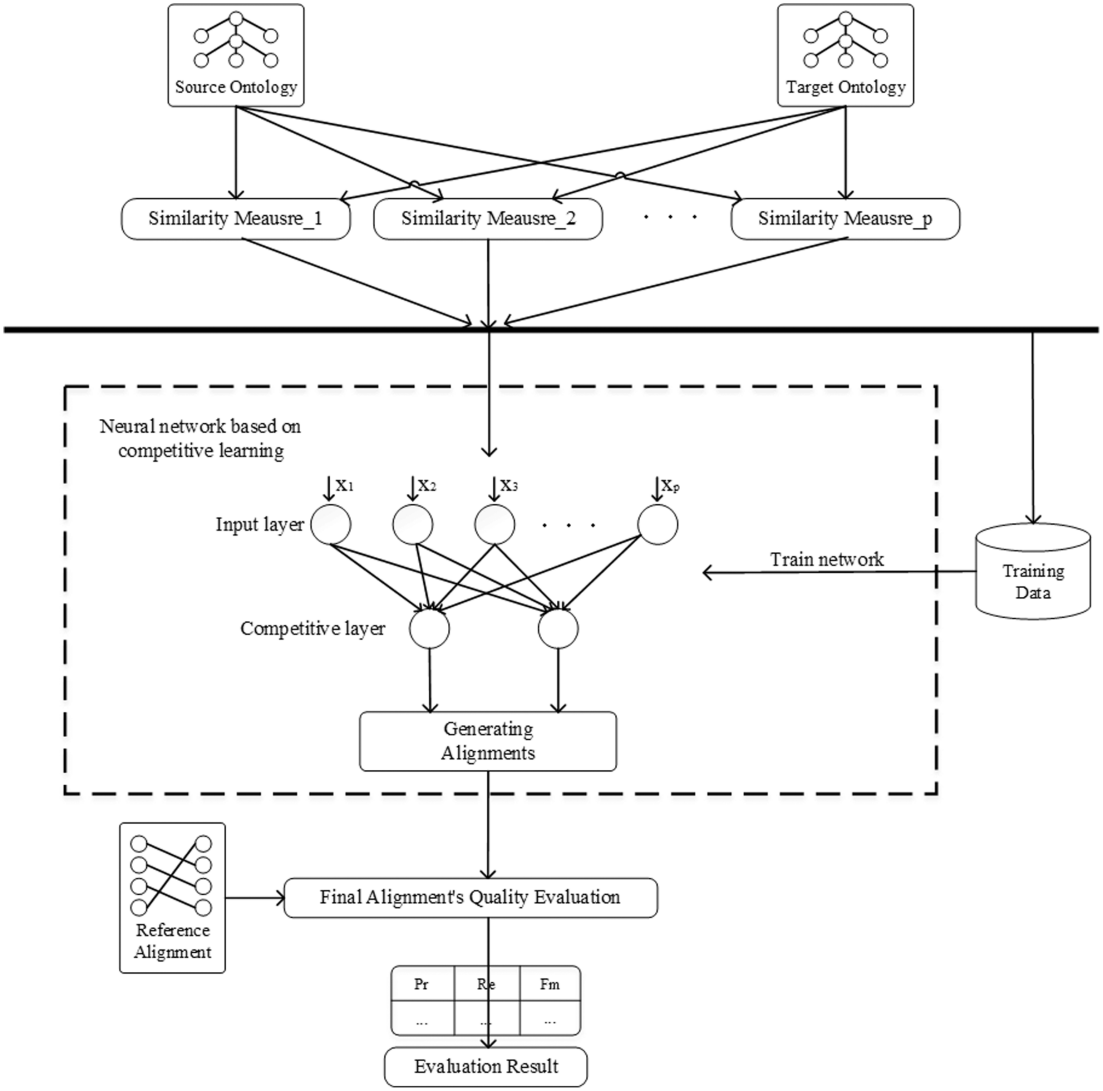
The framework of neural network based on competitive learning.

This section demonstrates the framework of the proposal, as shown in Fig. 3. First, extract representative entities from the ontologies to be aligned as the training samples and generate input using the similarity measures, which does not require manual labelling of training samples. In the hierarchy diagram of the ontology, all entities are sorted by the sum of their in-degree and out-degree, and then the entities among them are selected to construct the dataset. In this paper, 30% entities are randomly selected as the dataset. Then, train the network using competitive learning strategy and optimize the network weights. When the training of the network is finished, the remaining entity pairs to be matched are input to the network for classification to complete the ontology alignment. Finally, evaluate the final matching results using the reference alignment.

## Experiment

### Experimental configuration

In the experiments, the performance of our approach was tested using the benchmark track provided by the Ontology Alignment Evaluation Initiative (OAEI), as well as real sensor ontologies. Table 1 shows a detailed description of the benchmark test cases, where different series of test cases represent ontologies of different scales and domains. Select a representative case as the training dataset and the remaining cases of the same series are used as the test dataset. Table 2 presents a summary of the real sensor ontologies. Between the two sensor ontologies to be matched, thirty percent of the reference alignment is taken as the train dataset, and the remaining pairs are used as the test dataset. The recall and precision metrics are commonly used to evaluate the performance of matching systems in ontology alignment tasks, and the metrics are defined as follows:

**Table 1:** A detailed description of the benchmark test cases.

Cases ID	Cases introduction	Training case ID
101–104	Two same ontologies	#101
202–208	The ontologies have same structure, but different lexical and linguistic features	#208
221–247	The ontologies have the same lexical and linguistic features, but different structure	#221
248–262	The ontologies have different lexical, linguistic and structure features	#248

**Table 2:** A brief introduction to the real sensor ontologies.

Ontology name	Scale
Sensor, Observation, Sample and Actuator (SOSA) ontology^1^	42 entities
Semantic Sensor Network (SSN) ontology^2^	55 entities
SensorOntology2009 (SN) ontology^3^	152 entities
Original Semantic Sensor Network (OSSN) ontology^4^	107 entities

**Notes:**

1 https://www.w3.org/ns/sosa.

2 https://www.w3.org/ns/ssn.

3 https://www.w3.org/2005/Incubator/ssn/wiki/SensorOntology2009.

4 https://www.w3.org/2005/Incubator/ssn/wiki/Report_Work_on_the_SSN_ontology.

(7) $$precision = \displaystyle{{correct\_found\_correspondences} \over {all\_found\_correspondences}},$$

(8) $$recall = \displaystyle{{correct\_found\_correspondences} \over {all\_possible\_correspondences}}.$$

To harmonize precision and recall, we further use the f-measure, which is defined as follows:

(9) $$f\text{-}measure = \displaystyle{{2*recall*precision} \over {recall + precision}}.$$

### Comparison with OAEI’s participants

**Figure 4:**
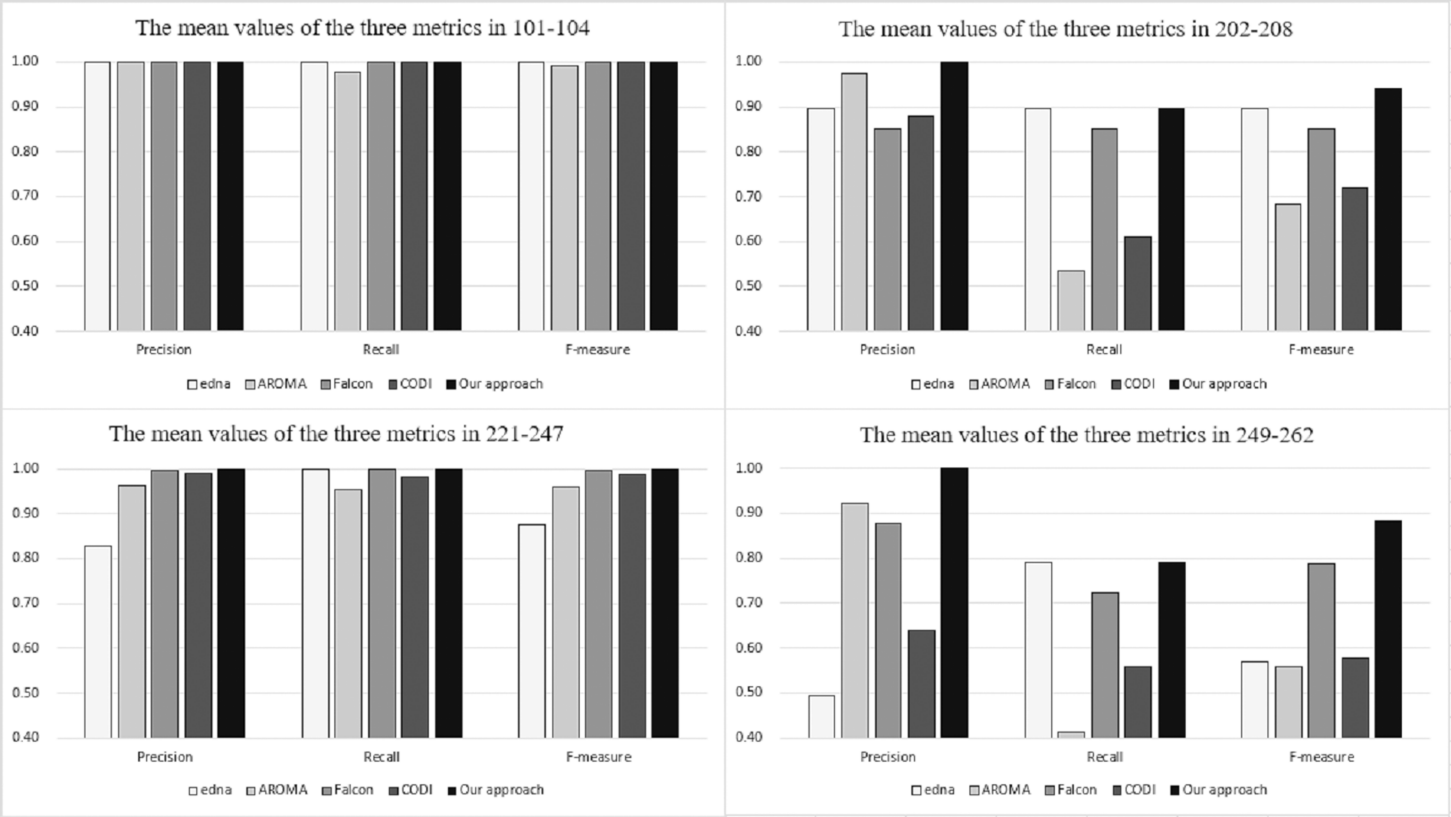
Comparison among our approach and OAEI’s participants.

Figure 4 depicts the comparison between our approach and OAEI’s participants in terms of precision, recall and f-measure. The relevant data are available from OAEI. The figure is divided into four sections corresponding to the experimental results for different series of test cases. In the figure, the vertical axis represents the alignment results, the horizontal axis represents the three evaluation metrics, and the legends represent the different matching systems. As can be seen from the figure, in the four series of test sets, our approach obtains better experimental results than the other matchers. Based on the experimental results, it can be concluded that our proposal is both effective and efficient. It is worth mentioning that the training cases do not need to be manually labelled with positive or negative samples, reducing a significant amount of work regarding pre-processing. In this respect, our approach outperforms other supervised matchers.

### Comparison with state-of-the-art ontology matchers

To verify the effectiveness of our approach in matching sensor ontologies, four popular ontology matching strategies were used as comparison groups, based on similarity flooding (SF) similarity (Boukhadra, Benatchba & Balla, 2015), Jaro–Winkler distance (Wang, Qin & Wang, 2017), Levenshtein distance (Behara, Bhaskar & Chung, 2020) and WordNet similarity (Beckwith et al., 2021), respectively. Table 3 exhibits the experimental results of our approach in terms of four sets of real sensor ontology alignment tasks and compares them with the results obtained by other strategies. As can be seen from the table, our approach outperforms the other matching strategies for most of the alignment tasks. In the “SSN-OSSN” task, our method is slightly lower than the Levenshtein distance and Jaro–Winkler distance. The reason is that individual alignment tasks can get better results using only a single similarity measure, and multiple measures become noisy. In addition, our strategy assumes that the alignment is one-to-one. Specifically, an entity in one ontology uniquely maps to another entity in another ontology. However, in some practical tasks, ontology mapping may be one-to-many or many-to-many. This can also explain that our strategy is lower than other strategies in terms of recall.

**Table 3:** Comparison among our approach and the state-of-the-art ontology matchers.

Alignment task	Ontology alignment metrics	SF-based matcher	Jaro–Winkler-based matcher	Levenshtein-based matcher	WordNet-based matcher	Our approach
	Recall	1.00	1.00	1.00	1.00	1.00
SOSA-SN	Precision	0.07	0.75	0.75	0.33	1.00
	f-measure	0.13	0.86	0.86	0.50	1.00
	Recall	0.50	1.00	1.00	1.00	1.00
SOSA-OSSN	Precision	0.20	1.00	1.00	0.67	1.00
	f-measure	0.29	1.00	1.00	0.80	1.00
	Recall	0.35	1.00	1.00	0.97	0.87
SSN-OSSN	Precision	0.06	0.94	1.00	0.80	1.00
	f-measure	0.11	0.97	1.00	0.88	0.93
	Recall	0.56	1.00	1.00	1.00	1.00
SSN-SN	Precision	0.02	0.90	1.00	0.52	1.00
	f-measure	0.04	0.95	1.00	0.70	1.00

## Conclusions

Sensor ontology matching technology can solve the problem of heterogeneity among sensor networks to ensure trusted communication and collaboration in AIoT. Different from traditional neural networks that require a large number of manually labelled training samples, this work proposes an unsupervised neural network to solve the ontology matching problem. The proposal first considers the ontology alignment task as a binary classification problem, then trains the network using a competitive learning strategy, and finally clusters the ontologies to be matched by the trained network. The experimental results present that the proposal obtains higher quality alignment results compared to other matchers in different domain knowledge such as biblio ontologies of OAEI and real sensor ontologies.

In the future, we are interested in using more similarity measures to determine the local similarity across the feature space and improve the ontology similarity results. In addition, we hope to map the relationships between ontology concepts to higher latitude feature spaces to obtain more accurate alignment results than just binary classification.

## Supplemental Information

10.7717/peerj-cs.763/supp-1 Supplemental Information 1 Raw data and code.

## Additional Information and Declarations

### Data Availability

The following information was supplied regarding data availability: The code for the experiments is available in the Supplementary File. The raw data are available at OAEI: http://oaei.ontologymatching.org/2016/results/benchmarks/index.html. The sensor ontologies are available at: - https://www.w3.org/ns/sosa - https://www.w3.org/ns/ssn - https://www.w3.org/2005/Incubator/ssn/wiki/SensorOntology2009 - https://www.w3.org/2005/Incubator/ssn/wiki/SSN\#Sensor.

## References

[ref-1] Alboukaey N, Joukhadar A (2018). Ontology matching as regression problem. Journal of Digital Information Management.

[ref-2] Anam S, Kim YS, Kang BH, Liu Q (2015). Review of ontology matching approaches and challenges. International Journal of Computer Science and Network Solutions.

[ref-3] Ardjani F, Bouchiha D, Malki M (2015). Ontology-alignment techniques: survey and analysis. International Journal of Modern Education & Computer Science.

[ref-4] Beckwith R, Fellbaum C, Gross D, Miller GA (2021). Wordnet: a lexical database organized on psycholinguistic principles.

[ref-5] Behara KN, Bhaskar A, Chung E (2020). A novel approach for the structural comparison of origin-destination matrices: Levenshtein distance. Transportation Research Part C: Emerging Technologies.

[ref-6] Bento A, Zouaq A, Gagnon M (2020). Ontology matching using convolutional neural networks.

[ref-7] Boukhadra A, Benatchba K, Balla A (2015). Similarity flooding for efficient distributed discovery of OWL-S process model in P2P networks. Procedia Computer Science.

[ref-8] Burhanuddin M, Mohammed AA-J, Ismail R, Hameed ME, Kareem AN, Basiron H (2018). A review on security challenges and features in wireless sensor networks: IoT perspective. Journal of Telecommunication, Electronic and Computer Engineering (JTEC).

[ref-9] Chen C-H (2020). An explainable deep neural network for extracting features. https://science.sciencemag.org/content/365/6452/416/tab-e-letters.

[ref-10] Chen C-H, Song F, Hwang F-J, Wu L (2020). A probability density function generator based on neural networks. Physica A: Statistical Mechanics and Its Applications.

[ref-11] Chu S-C, Xue X, Pan J-S, Wu X (2020). Optimizing ontology alignment in vector space. Journal of Internet Technology.

[ref-12] Djeddi WE, Khadir MT (2012). Introducing artificial neural network in ontologies alignment process. Control and Cybernetics.

[ref-13] Ghosh A, Chakraborty D, Law A (2018). Artificial intelligence in internet of things. CAAI Transactions on Intelligence Technology.

[ref-14] Hariri BB, Abolhassani H, Sayyadi H (2006). A neural-networks-based approach for ontology alignment.

[ref-15] Huang Y, Xue X, Jiang C (2020). Semantic integration of sensor knowledge on artificial internet of things. Wireless Communications and Mobile Computing.

[ref-16] Jiang C, Xue X (2020). Matching biomedical ontologies with long short-term memory networks.

[ref-17] Jiang C, Xue X (2021). A uniform compact genetic algorithm for matching bibliographic ontologies. Applied Intelligence.

[ref-18] Khoudja MA, Fareh M, Bouarfa H (2018a). A new supervised learning based ontology matching approach using neural networks.

[ref-19] Khoudja MA, Fareh M, Bouarfa H (2018b). Ontology matching using neural networks: survey and analysis.

[ref-20] Kocakulak M, Butun I (2017). An overview of wireless sensor networks towards internet of things.

[ref-21] Li W-S, Clifton C (2000). SEMINT: a tool for identifying attribute correspondences in heterogeneous databases using neural networks. Data & Knowledge Engineering.

[ref-22] Li S, Xu LD, Zhao S (2015). The internet of things: a survey. Information Systems Frontiers.

[ref-23] Lin JC-W, Srivastava G, Zhang Y, Djenouri Y, Aloqaily M (2020). Privacy-preserving multiobjective sanitization model in 6G IoT environments. IEEE Internet of Things Journal.

[ref-24] Liu J, Li Y, Tian X, Sangaiah AK, Wang J (2019). Towards semantic sensor data: an ontology approach. Sensors.

[ref-25] Lv Q, Jiang C, Li H (2020). Solving ontology meta-matching problem through an evolutionary algorithm with approximate evaluation indicators and adaptive selection pressure. IEEE Access.

[ref-26] Rubiolo M, Caliusco ML, Stegmayer G, Coronel M, Fabrizi MG (2012). Knowledge discovery through ontology matching: an approach based on an artificial neural network model. Information Sciences.

[ref-27] Sisinni E, Saifullah A, Han S, Jennehag U, Gidlund M (2018). Industrial internet of things: challenges, opportunities, and directions. IEEE Transactions on Industrial Informatics.

[ref-28] Sung T-W, Yang C-S (2013). Distributed voronoi-based self-redeployment for coverage enhancement in a mobile directional sensor network. International Journal of Distributed Sensor Networks.

[ref-29] Sung T-W, Yang C-S (2014). Voronoi-based coverage improvement approach for wireless directional sensor networks. Journal of Network and Computer Applications.

[ref-30] Wang Y, Qin J, Wang W (2017). Efficient approximate entity matching using jaro-winkler distance.

[ref-31] Wang X, Zhang X, Li M (2015). A survey on semantic sensor web: sensor ontology, mapping and query. International Journal of u- and e-Service, Science and Technology.

[ref-32] Xu Y, Helal A (2015). Scalable cloud-sensor architecture for the internet of things. IEEE Internet of Things Journal.

[ref-33] Xue X (2020). A compact firefly algorithm for matching biomedical ontologies. Knowledge and Information Systems.

[ref-34] Xue X, Chen J (2018). A preference-based multi-objective evolutionary algorithm for semiautomatic sensor ontology matching. International Journal of Swarm Intelligence Research (IJSIR).

[ref-35] Xue X, Chen J (2019). Using compact evolutionary Tabu search algorithm for matching sensor ontologies. Swarm and Evolutionary Computation.

[ref-36] Xue X, Chen J (2020). Optimizing sensor ontology alignment through compact co-firefly algorithm. Sensors.

[ref-37] Xue X, Liu J (2017). Collaborative ontology matching based on compact interactive evolutionary algorithm. Knowledge-Based Systems.

[ref-38] Xue X, Pan J-S (2018). A compact co-evolutionary algorithm for sensor ontology meta-matching. Knowledge and Information Systems.

[ref-39] Xue X, Wang Y (2015). Optimizing ontology alignments through a memetic algorithm using both matchFmeasure and unanimous improvement ratio. Artificial Intelligence.

[ref-40] Xue X, Wu X, Jiang C, Mao G, Zhu H (2021a). Integrating sensor ontologies with global and local alignment extractions. Wireless Communications and Mobile Computing.

[ref-41] Xue X, Yang C, Jiang C, Tsai P-W, Mao G, Zhu H (2021b). Optimizing ontology alignment through linkage learning on entity correspondences. Complexity.

[ref-42] Xue X, Zhang J (2021). Matching large-scale biomedical ontologies with central concept based partitioning algorithm and adaptive compact evolutionary algorithm. Applied Soft Computing.

[ref-43] Zhu H, Zhang J, Xue X (2021). Semisupervised learning-based sensor ontology matching. Security and Communication Networks.

